# Validity issues in measures of COVID-19 preventive behaviours

**DOI:** 10.7189/jogh.13.03026

**Published:** 2023-06-09

**Authors:** Junhua Dang, Lile Jia

**Affiliations:** 1 School of Psychology, Beijing Sport University, Beijing, China; 2 Department of Surgical Sciences, Uppsala University, Uppsala, Sweden; 3 Department of Psychology, National University of Singapore, Singapore; 4 Center for Population Health, National University of Singapore, Singapore

The global coronavirus disease 2019 (COVID-19) outbreak has become the most severe crisis in recent history. The virus spreads primarily through droplets people send out when they talk, sneeze, or cough. Therefore, preventive behaviours such as mask-wearing, social distancing, and movement restraint are integral to public health campaigns against the COVID-19 pandemic. Accordingly, scientists have investigated community- or individual-level predictors of preventive behaviours through either indirect or direct measuring approaches. Indirectly, researchers used morbidity/mortality as an inverse proxy for preventive behaviours, assuming low morbidity/mortality as an outcome of more preventive behaviours. Directly, researchers either collected people’s self-reports of preventive behaviours or employed objective measures such as mobility trends recorded by mobile phones.

No measurement, however, is perfect. Here we discuss the validity issues in the three measurement types by scrutinising the extent to which they represent what they are supposed to capture (i.e. preventive behaviours) and provide recommendations on how to best utilise these measures (**Table 1**).

**Table 1:** Comparing the three measurement types of preventive behaviours

Measurement type	Validity issues	Recommendations
Morbidity/mortality	Inaccurate and varied inaccuracy across countries	Officially reported data may be considered within a specific country. Excess mortality may be comparable across countries.
Self-reports	Contaminated by social desirability	Should be interpreted with caution and best combined with objective measures
Objective measures	Accurate; need to be interpreted carefully	Consider contextual factors and develop measures indicative of mask-wearing and physical hygiene

## VALIDITY ISSUES

First, officially reported morbidity/mortality is highly inaccurate. For morbidity, the difficulty in detecting asymptomatic infections and limitations due to varying testing capacity between countries has likely resulted in substantial underreporting of cases. For instance, it was estimated that only 1.4% of infections in Africa were reported [1]. Mortality data do not fare better. Based on data from large-scale surveys, excess deaths, burials, cemeteries, crematoriums, and hospital mortuaries, recent studies estimated that officially reported COVID-19 deaths represented only a portion of actual deaths [2], ranging from 75% in Europe [3] to only 12.5% in India [4]. The issue with reporting inaccuracy is not unique to COVID-19 but is common in most infectious disease outbreaks. For instance, the estimated deaths caused by malaria worldwide were four times higher than official statistics [5]. A retrospective analysis based on records of burials during the 2014-2016 Ebola epidemic in Sierra Leone revealed that the number of estimated deaths deviated significantly from that of reported cases, especially in regions far from the capital district [6]. This problem has not received adequate scholarly attention. The wide variation in inaccuracy prevents any cross-country comparisons. One study, for instance, found that, during the COVID-19 pandemic, regions with tight cultures and countries with stricter rules and punishments for deviance (e.g. Singapore and South Korea), had fewer reported infections and deaths than regions with loose cultures and countries that have weaker norms and were more permissive for atypical behaviour (e.g. Brazil and Spain) [7]. While the researchers concluded that the strong social norms in tight cultures encouraged collective, preventive behaviours, the results could also have come from the cross-country inaccuracies in morbidity/mortality.

Second, self-reports suffer severely from social desirability bias, as respondents tend to report an inflated degree of adherence to preventive behaviours. For instance, in a large-scale worldwide survey (n = 49 968 across 67 countries), most participants reported complete compliance with all possible preventions. The global means of following guidelines for social distancing and physical hygiene were improbably high, at 8.60 and 8.21, respectively (scale range = 0 (strongly disagree) to 10 (strongly agree)) [8]. Similar patterns have also been found during the 2014-2016 Ebola epidemic. Almost all participants indicated that they washed hands nearly every time (mean = 4.51; scale range = 1 (never) to 5 (every time)) [9]. Statistically, the highly skewed data restricts the variability in people’s responses, greatly reducing statistical power. Conceptually, because people report their behaviours in a socially desirable manner (i.e. to be perceived as a good citizen), self-report data could substantially distort the true relationship between relevant factors and preventive behaviours.

Finally, although objective measures are more accurate than morbidity/mortality and self-reports, whether they can represent preventive behaviours is unclear. For example, researchers consider high mobility as evidence of noncompliance with preventive guidelines. However, people may move a lot to conduct daily activities (e.g. grocery shopping) while strictly complying with relevant rules (e.g. mask-wearing, two-meter social distancing). Therefore, such data must be interpreted with caution.

## RECOMMENDATIONS

Morbidity/mortality should only be used to compare communities covered by the same political and health system. Though inaccuracy may still exist, the bias in reporting should be unidirectional and otherwise similar, allowing for the association between community-level predictors and preventive behaviours to be estimated. Further, researchers should consider excess mortality a better indicator than officially reported deaths [2]; this variable is now tracked by the World Mortality Data set [10].

Self-reports should only be used with large sample sizes, which helps alleviate statistical concerns (i.e. restricted variability). Conceptual concerns (i.e. indicative of impression management rather than preventive behaviours) are more problematic. It is best to cross-check self-reports and objective measures for convergent evidence. For example, collectivism has been associated with both self-reports of obeying shelter-in-place orders and mobility trends indicative of staying-at-home duration [11]. In such cases, we can be more confident of our conclusions despite the validity issues in both measures.

Objective measures’ validity may depend on such contextual factors as policy stringency. For example, during the full lockdown period, mobility trends public spaces are a good indicator of non-compliance with preventive guidelines. However, when the lockdown is only partially or loosely implemented, mobility trends alone can be seriously biased without corroborating evidence (e.g. images or videos). Unfortunately, objective data reflecting other preventive behaviours like mask-wearing and physical hygiene are less available. Therefore, mobility data need more nuanced analysis and data from other sources need to be further developed.

## CONCLUSION

Research on preventive behaviours during the COVID-19 pandemic showcases the importance of behavioural science. However, scholars’ enthusiasm for uncovering societally impactful findings must be matched with earnest scrutiny of their methodology. We call for some healthy reflection in our work.
